# FGF19/FGFR4 signaling contributes to the resistance of hepatocellular carcinoma to sorafenib

**DOI:** 10.1186/s13046-016-0478-9

**Published:** 2017-01-09

**Authors:** Lixia Gao, Xuli Wang, Yaoliang Tang, Shuang Huang, Chien-An Andy Hu, Yong Teng

**Affiliations:** 1 Department of Oral Biology, Dental College of Georgia, Augusta University, 1120 15th Street, Augusta, GA 30912 USA; 2 Department of Radiology and Imaging Sciences, School of Medicine, University of Utah, Salt Lake City, UT USA; 3 Experimental Therapeutics Program, Huntsman Cancer Institute, University of Utah, Salt Lake City, UT USA; 4 Vascular Biology Center, Department of Medicine, Medical College of Georgia, Augusta University, Augusta, GA USA; 5 Department of Anatomy and Cell Biology, University of Florida College of Medicine, Gainesville, FL USA; 6 Department of Biochemistry and Molecular Biology, University of New Mexico School of Medicine, Albuquerque, NM USA; 7 Department of Biochemistry and Molecular Biology, Medical College of Georgia, Augusta University, 1120 15th Street, Augusta, GA USA

**Keywords:** FGF19, FGFR4, Hepatocellular carcinoma, Drug resistance, Sorafenib, Synergistic effect

## Abstract

**Background:**

Sorafenib, a multi-kinase inhibitor, is used as a standard therapy for advanced hepatocellular carcinoma (HCC). However, complete remission has not been achieved and the molecular basis of HCC resistance to sorafenib remains largely unknown. Previous studies have shown that fibroblast growth factor 19 (FGF19) expression correlates with tumor progression and poor prognosis of HCC. Here, we demonstrate the novel role of FGF19 in HCC resistance to sorafenib therapy.

**Methods:**

FGF19 Knockdown cells were achieved by lentiviral-mediated interference, and FGFR4 knockout cells were achieved by CRISPR-Cas9. Protein levels of FGF19, FGFR4 and c-PARP in various HCC cell lines were measured by Western blotting analysis. Cell viability was determined by MTS assay, apoptosis was determined by DAPI nuclear staining and Western blot of c-PRAP, and ROS generation was determined by DCFH-DA staining and electrochemical biosensor.

**Results:**

We showed that FGF19, when overexpressed, inhibited the effect of sorafenib on ROS generation and apoptosis in HCC. In contrast, loss of FGF19 or its receptor FGFR4 led to a remarkable increase in sorafenib-induced ROS generation and apoptosis. In addition, knockdown of FGF19 in sorafenib-resistant HCC cells significantly enhanced the sensitivity to sorafenib. Importantly, targeting FGF19/FGFR4 axis by ponatinib, a third-generation inhibitor of chronic myeloid leukemia, overcomes HCC resistance of sorafenib by enhancing ROS-associated apoptosis in sorafenib-treated HCC.

**Conclusion:**

Our results provide the first evidence that inhibition of FGF19/FGFR4 signaling significantly overcomes sorafenib resistance in HCC. Co-treatment of ponatinib and sorafinib may represent an effective therapeutic approach for eradicating HCC.

**Electronic supplementary material:**

The online version of this article (doi:10.1186/s13046-016-0478-9) contains supplementary material, which is available to authorized users.

## Background

Hepatocellular carcinoma (HCC) is the sixth common malignancies worldwide and the third leading cause of cancer-associated mortality [1–5]. Although advances in diagnostic techniques and instrumentation of oncology have improved the early diagnosis of HCC, the median survival of patients with this disease is still low. Recently, a number of molecular targeted drugs have been illustrated to be promising agents in prolonging the overall survival of patients with advanced HCC. Particularly, as a multikinase inhibitor of Raf/MEK/ERK signaling and the receptor tyrosine kinases (RTKs), sorafenib leads to a survival benefit for patients through reducing tumor angiogenesis and increasing cancer cell apoptosis [6–9]. However, its use is often hampered by the occurrence of drug resistance [10–12]. Urgently needed to resolve the problem is to explore the mechanisms of resistance on sorafenib and seek an effective systemic therapy for patients after failure of sorafenib treatment.

FGF19 is a metabolic regulator gene belonging to the hormone-like FGF family of signal molecules, and has activity as an ileum-derived postprandial hormone [13, 14]. Genomic and functional analyses show that FGF19 acts as an oncogenic driver in HCC [15–17]. FGFR4 is the predominant FGFR isoform in FGFRs in human hepatocytes and both FGF19 and FGFR4 are highly expressed in primary HCC [18]. FGF19 has unique specificity for FGFR4 [19], and through binding to it, FGF19 activates different intracellular pathways, including GSK3β/β-catenin/E-cadherin signaling [20]. Emerging studies indicate a focal, high-level amplification frequency of FGF19 in HCC clinical samples, which is positively correlated with tumor size, pathological stage and poor prognosis [15, 21–23]. Recently, HCC responder cases to sorafenib were collected to explore the association between the efficacy of sorafenib and gene alterations [24]. Using next generation sequencing and copy number assay, an FGF19 copy number gain was detected more frequently among complete response cases than among non-complete response cases, suggesting FGF19 amplification may be a predictor of a response to sorafenib [24]. Therefore, increased understanding of the clinical relevance of FGF19 may bring molecular insights into the pathogenesis and treatment of HCC.

In this work, we determined the importance of FGF19 in sorafenib-induced cell viability, apoptosis, and accumulation of mitochondrial reactive oxidative species (ROS). We also evaluated the role of FGF19 and FGF19/FGFR4 axis in sorafenib resistance, and determined the synergistic effect of sorafenib and FGFR inhibitor ponatinib on sorafenib-resistant HCC cells. Our data reveal that FGF19 is essential for sorafenib efficacy and resistance in the treatment of HCC. This study provides critical rationale to test the inhibition of FGF19 signaling in patients with sorafenib-resistant HCC.

## Methods

### Cell lines, reagents and standard assays

HCC cell lines (MHCC97L, MHCC97H, HepG2, and SMMC7721) were directly obtained from American Type Culture Collection (ATCC, Rockville, MD). Sorafenib and ponatinib were purchased from Selleckchem (Houston, TX, USA). Superoxide dismutase (SOD), DMSO and DAPI were purchased from Sigma-Aldrich (St. Louis, MO). Standard cell culture, transient transfections, lentiviral transduction, quantitative RT-PCR (qRT-PCR), western blot, and cell viability assays were carried out as described previously [20].

### Antibodies and constructs

Antibodies raised against FGF19 and FGFR4 were purchased from Abcam (Cambridge, MA), β-actin was from Sigma-Aldrich (St Louis, MO), and cleaved PAPR (c-PARP) was from Cell Signaling (Beverly, MA). The full-length of human FGF19 and FGFR4 cDNA were cloned into pcDNA3.1(+) expression vector (Life technologies, Carlsbad, CA). Lentiviral vectors harboring shRNAs targeting FGF19 were obtained from GeneCopoeia (Rockville, MD). LentiCRISPR v2 vector used for generating CRISPR-Cas9 targeted deletion of FGFR4 was obtained from Feng Zhang (Addgene plasmid #52961). All the plasmids used in this study were verified by sequencing.

### Development of sorafenib resistant cells

To generate sorafenib-resistant cells, cells were treated with LC50 of sorafenib and the concentration was gradually increased by 10% every 2 weeks until the maximum tolerated doses (10 μM) have been reached. Sorafenib-resistant cells were continuously cultured in the presence of 1 μM of sorafenib.

### Electrochemical detection of O_2_^•-^

Electrochemical detection of superoxide (O_2_^**•-**^) released from cells was established as previously described [25]. In brief, 5 × 10^5^ cells were incubated with sorafenib or/and other regents as indicated. A cyclic voltammetry (CV) was used to monitor cellular O_2_^•-^ generation on CHI760E electrochemical station (ChenHua Instruments, Wuhan, China). SOD was added to the medium to verify the current changes was caused by O_2_^•-^. The electrochemical sensors were calibrated at different concentrations of O_2_^•-^ in a fluidic chamber, and percentages of peak (potential = 0.7 V; current enhancement) were compared and calculated against the control curve and evaluated the release of the analysts.

### Fluorescence analysis of intracellular oxidative stress

To further validate the generation of O_2_^•-^, intracellular ROS were also determined by the oxidant-sensing fluorescent probe 2′,7′-dichlorodihydrofluorescein diacetate (DCFH-DA, Sigma-Aldrich). Briefly, cells were incubated with 10 μM of DCFH-DA for 20 min at 37 °C, after which they were washed, trypsinized, resuspended and immediately analyzed for fluorescence intensity under a fluorescence microscope (IX-71, Olympus Corp., Tokyo, Japan). Median fluorescence intensity was quantified by the NIH ImageJ software.

### Determination of apoptotic bodies by DAPI nuclear staining

The presence of apoptotic bodies and nuclei morphology were determined by DAPI staining. Briefly, cells were fixed in 4% paraformaldehyde-PBS solution for 10 min and were stained with DAPI (300 nM) for 30 min at room temperature. Cells were examined for apoptotic bodies and nuclear morphology and photographed under fluorescence microscopy. Apoptotic cells were recognized and determined based on characteristic observations including the presence of fragmented, condensed, and degraded nuclei.

### Statistical analysis

The data were presented as means ± SD from three or more independent experiments and were analyzed by the Student’s *t*-test at a significance level of *P* < 0.05.

## Results

### Sorafenib induces ROS-associated apoptosis in HCC cells

**Fig. 1:**
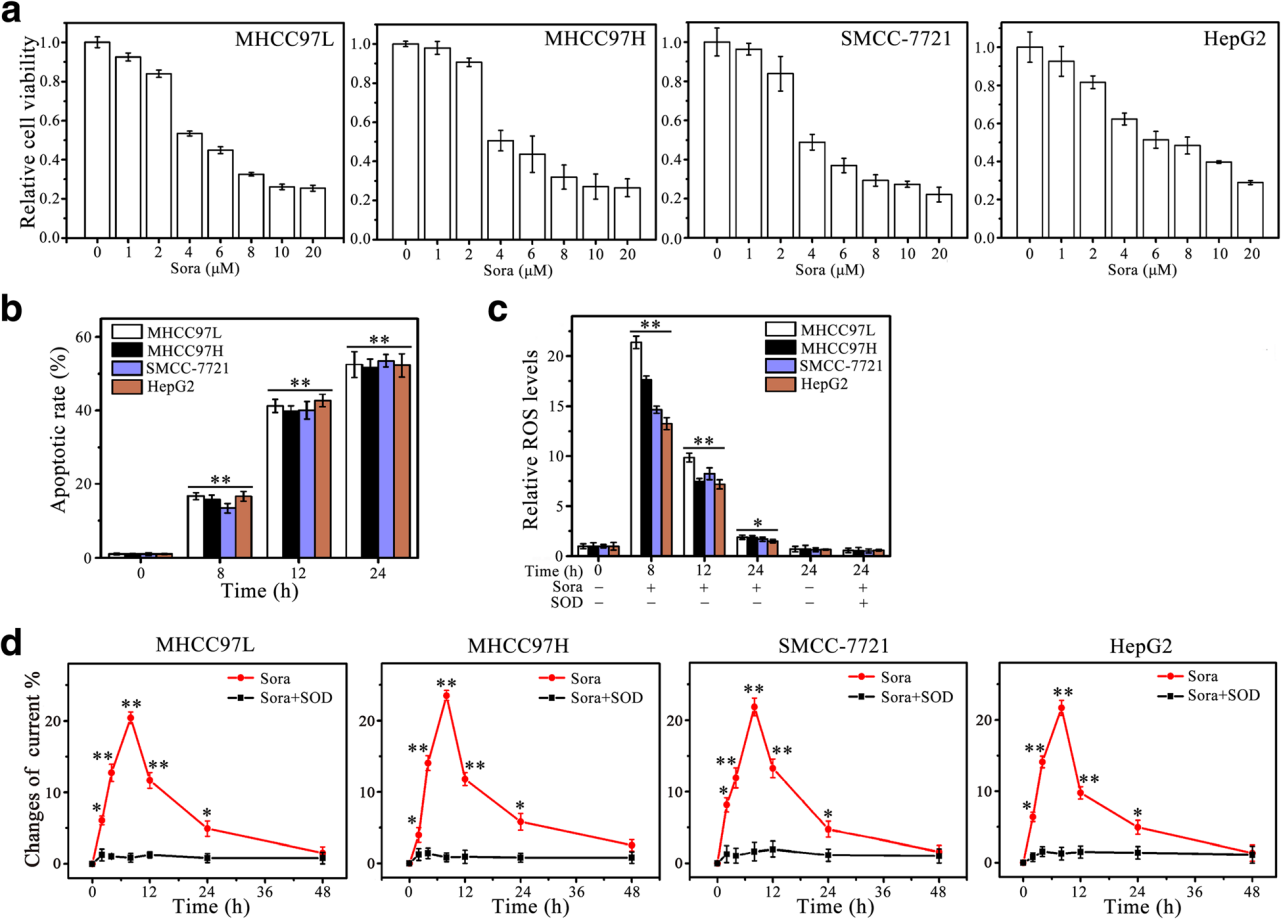
Sorafenib induces apoptosis and ROS generation in HCC cells. **a** The effect of sorafenib (Sora) on cell viability. The HCC cell lines (MHCC97L, MHCC97H, HepG2 and SMCC-7721) were treated with indicated concentrations of Sora for 24 h, and cell viability was determined by MTS assays as previously described. **b–d** The effect of Sora on cell apoptosis and ROS generation. The HCC cell lines were treated with Sora (4 μM for MHCC97L, MHCC97H and SMCC-7721, and 6 μM for HepG2) over a series of time points. Apoptosis was determined by DAPI staining (**b**); ROS generation was determined by DCFH-DA staining (**c**); and O_2_^•−^ generation was determined by electrochemical biosensor (**d**). SOD: superoxide dismutase. * *p* < 0.05; ** *p* < 0.01

To determine the effective dose range of sorafenib in human HCC cells, four cell lines (MHCC97L, MHCC97H, HepG2 and SMCC7721) were selected and treated with varied doses of sorafenib. The average LI50 (Lethal Concentration 50%) in MHCC97L, MHCC97H and SMCC7721 cells is ~4 μM, while HepG2 cells have LC50 value of 6 μM (Fig. 1a). We used the LC50 dosages to treat different HCC cell lines and determined the effect of sorafenib on apoptosis. Results from DAPI staining showed that sorafenib induced ~16, 40 and 52% apoptotic cells in MHCC97L cells at 8, 12, 24 h after treatment, respectively (Fig. 1b and Additional file 1: Figure S1A). The similar apoptotic response was observed in other three cell lines (Fig. 1b and Additional file 1: Figure S1A).

ROS and the resulting oxidative stress play a pivotal role in apoptosis [26, 27]. We thus investigated the relationship between sorafenib-induced apoptosis and oxidative stress. ROS production was evaluated by DCFH-DA, showing a significant increase of fluorescence in the cells exposed to sorafenib (Fig. 1c and Additional file 1: Figure S1B). These observations suggest that sorafenib-induced oxidative stress may lead to HCC cell apoptosis. To study whether the oxidative stress induced by sorafenib was mediated via O_2_^•−^, we monitored O_2_^•−^ release by the well-established electrochemical biosensors (Additional file 2: Figure S2). The amount of O_2_^•−^ generation (a 25% increase compared with the controls) in all examined cell lines reached maximum at 8 h after sorafenib treatment and then decreased in the following treatment (Fig. 1d), which was consistent with the trend of ROS generation in presence of sorafenib (Fig. 1c). These data suggest that elevated intracellular O_2_^•−^ levels contribute to sorafenib-induced oxidative stress.

### FGF19 is a critical regulator involved in HCC cell response to sorafenib

**Fig. 2:**
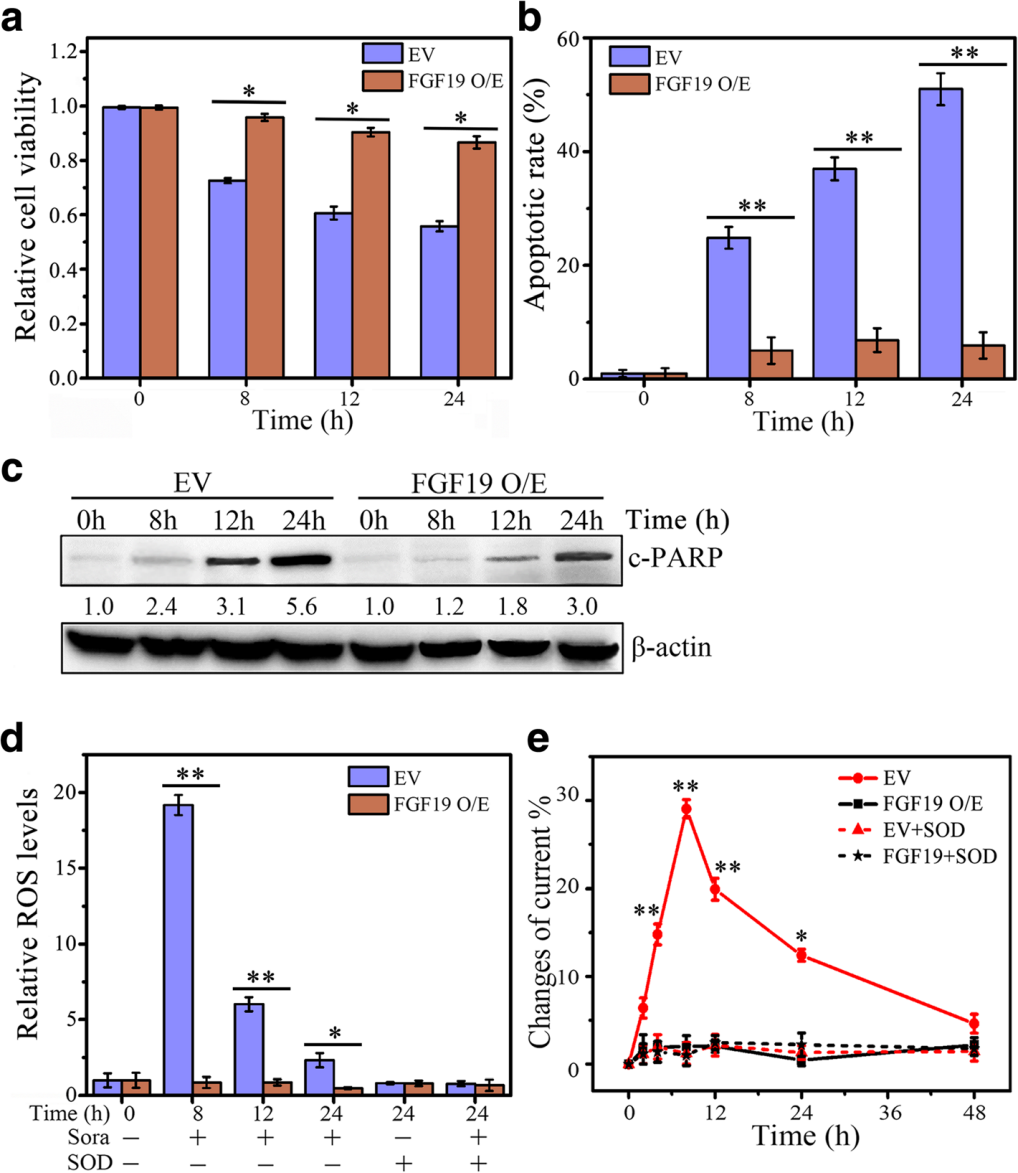
FGF19 overexpression protects HCC cells against sorafenib. **a**–**e** The effect of FGF19 overexpression on Sora-induced HCC cell apoptosis and ROS generation. MHCC97L cells expressing pcDNA3.1-FGF19 (FGF19 O/E) or empty vector (EV) were treated with 4 μM of Sora over a series of time points before analysis. Cell viability was determined by MTS assays (**a**); cell apoptosis was determined by DAPI staining (**b**) and Western blot of cleaved PARP (c-PARP) (**c**); ROS generation was determined by DCFH-DA staining (**d**); and O_2_^•−^ generation was determined by electrochemical biosensor (**e**). In (**c**), expression levels were normalized against actin and reported relative to controls (fold changes shown below each lane). * *p* < 0.05; ** *p* < 0.01

To investigate the impact of FGF19 on sorafenib-induced HCC cell apoptosis and ROS generation, we manipulated FGF19 expression levels in different HCC cell lines. Cell viability assays showed that FGF19 overexpression in MHCC97L cells prevented cell death against sorafenib (Fig. 2a). Increased apoptotic cells and cleaved PARP were also seen in the sorafenib treatment, which were attenuated when FGF19 was overexpressed (Fig. 2b and c). Moreover, forced expression of FGF19 abolished sorafenib-induced production of ROS and O_2_^•−^ (Fig. 2d and e).

**Fig. 3:**
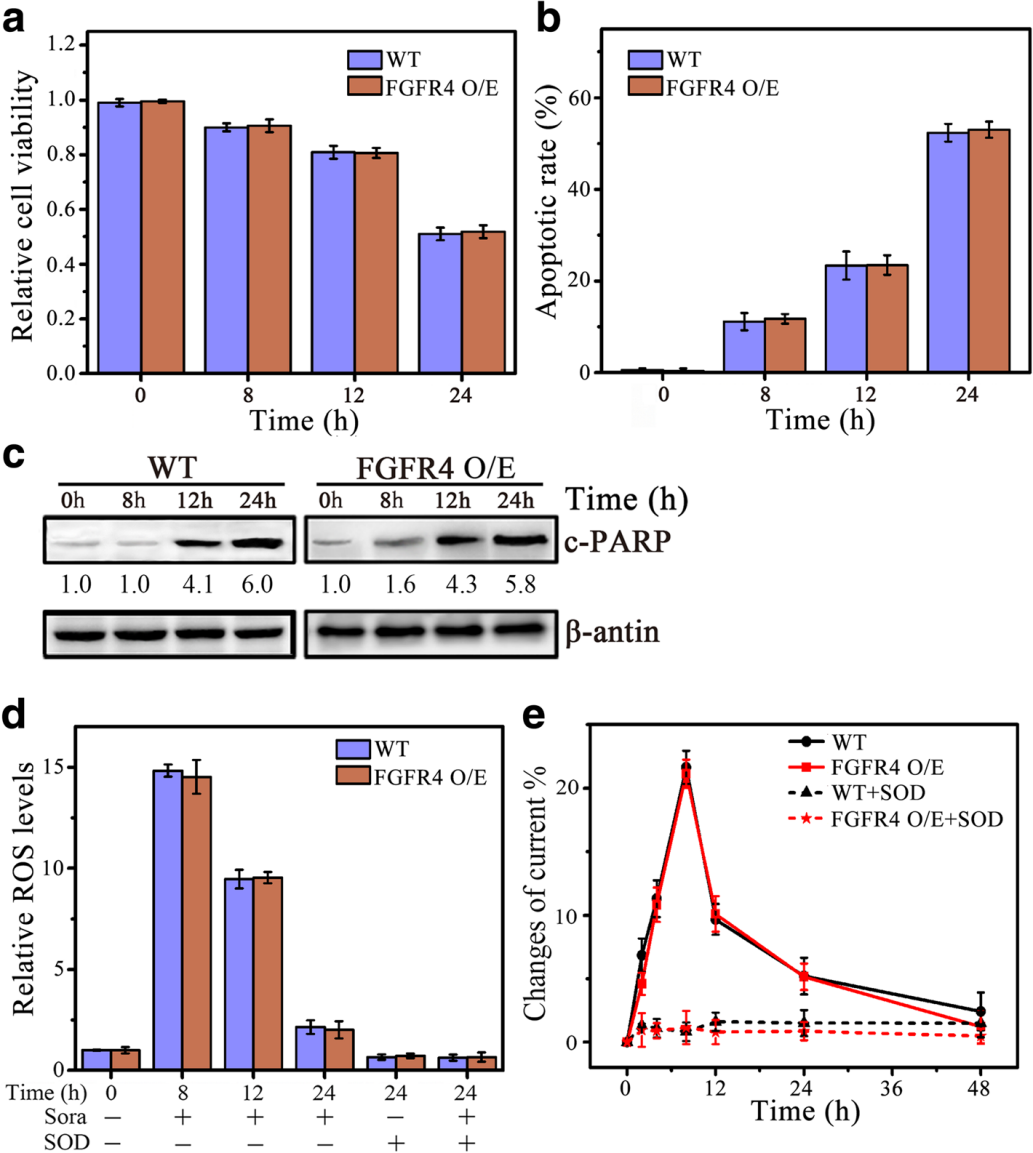
Loss of FGF19 expression enhances sorafenib-induced apoptosis associated with ROS. **a**–**e** The effect of FGF19 depletion on Sora-induced HCC cell apoptosis and ROS generation. MHCC97H cells expressing FGF19 shRNA (shFGF19) or control shRNA (shNC) were treated with 4 μM of Sora over a series of time points before analysis. Cell viability was determined by MTS assays (**a**); apoptosis was determined by DAPI staining (**b**) and Western blot of c-PRAP (**c**); ROS generation was determined by DCFH-DA staining (**d**); and O_2_^•−^ generation was determined by electrochemical biosensor (**e**). In **c**, expression levels were normalized against actin and reported relative to controls (fold changes shown below each lane). * *p* < 0.05; ** *p* < 0.01

We next examined the effects of FGF19 knockdown on sorafenib-induced cell phenotypes. As expected, depletion of FGF19 in MHCC97H cells significantly decreased survival cells exposed to sorafenib (Fig. 3a). FGF19 knockdown also enhanced apoptotic rate in the treatment of sorafenib (Fig. 3b and c), showing increased cells with apoptotic nuclei and increased cleaved PARP levels. In contrast to the phenotypes observed in FGF19 overexpressing cells, loss of FGF19 expression enhanced ROS generation and O_2_^•−^ release when compared with the knockdown control cells (Fig. 3d and e). Collectively, these observations demonstrate FGF19 is deeply involved in sorafenib-induced cell response.

### FGFR4 is essential for ROS-associated apoptosis by sorafenib

**Fig. 4:**
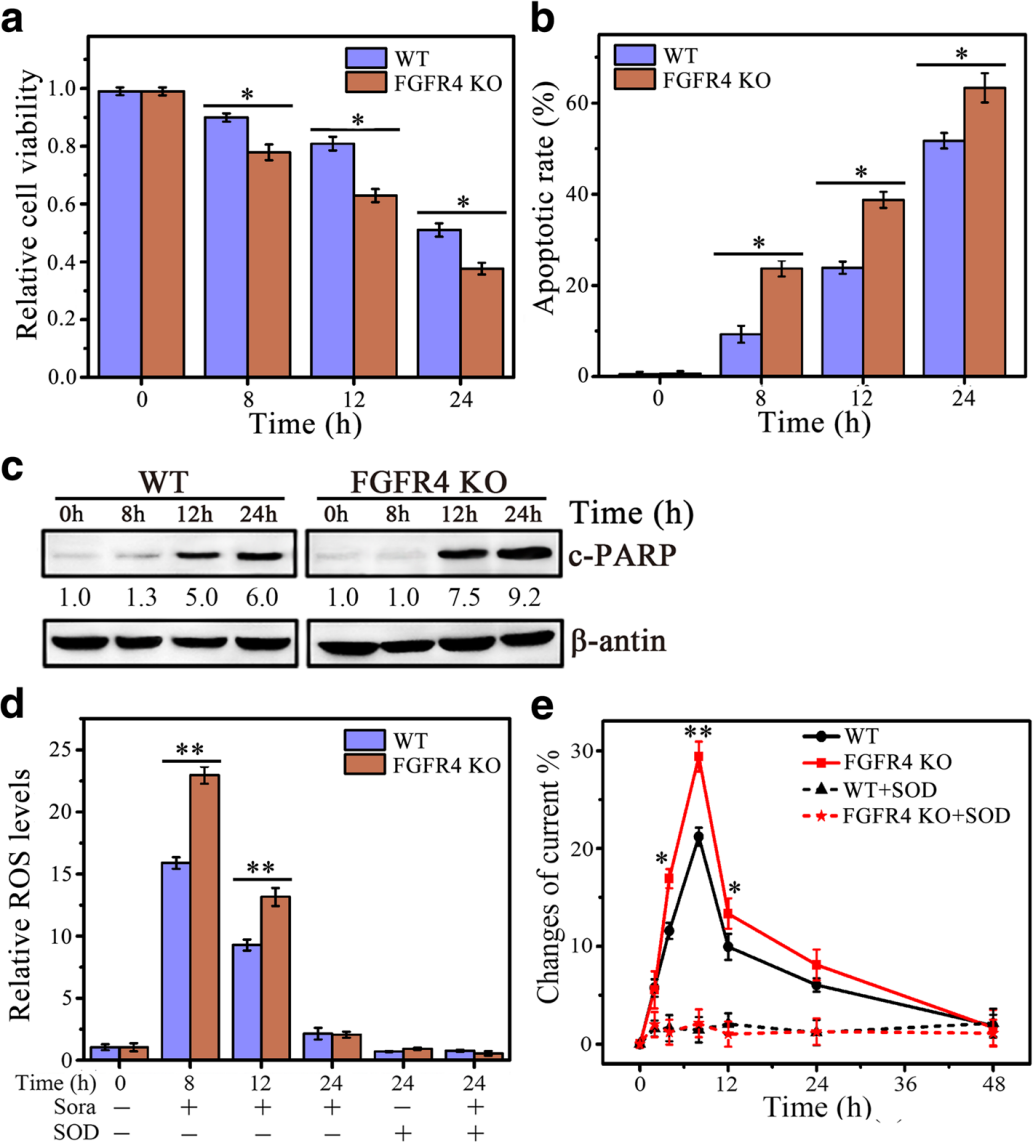
Knockdown of FGFR4 promotes ROS-associated HCC cell apoptosis by sorafenib. **a**–**e** The effect of FGFR4 knockout on Sora-induced HCC cell apoptosis and ROS generation. FGFR4 knockout MHCC97L cells by CRISPR sgRNA targeting exonic regions (FGFR4 KO) were treated with 4 μM of Sora over a series of time points before analysis. Cell viability was determined by MTS assays (**a**); Apoptosis was determined by DAPI staining (**b**) and Western blot of c-PARP (**c**); ROS generation was determined by DCFH-DA staining (**d**); and O_2_^•−^ generation was determined by electrochemical biosensor (**e**). In **c**, expression levels were normalized against actin and reported relative to controls (fold changes shown below each lane). * *p* < 0.05; ** *p* < 0.01

FGF19 drives cell proliferation and migration through interacting with its cognate receptor FGFR4 [28], which promoted us to investigate the importance of FGFR4 in sorafenib-induced cell apoptosis. Similar to loss of FGF19 expression (Fig. 3), FGFR4 knockout by CRISPR-Cas9 system decreased cell survival of MHCC97H in response to sorafenib (Fig. 4a), and enhanced cell apoptosis by sorafenib accompanying increased generation of ROS and O_2_^•−^ (Fig. 4b–e). Interestingly, overexpression of FGFR4 in MHCC97L cells did not produce any effect on cell viability, apoptosis and ROS release when exposed to sorafenib (Additional file 3: Figure S3). These data demonstrate that the role of FGFR4 in sorafenib is largely dependent on FGF19 status, suggesting FGF19/FGFR4 axis plays an essential part in the resistance of HCC cells to sorafenib.

### Loss of FGF19 in sorafenib-resistant HCC cells increases the sensitivity to sorafenib

To study the mechanism of sorafenib resistance, we generated sorafenib-resistant HCC cells. MHCC97H cells displayed a spindle-shaped appearance, whereas these cells experiencing long-term administration of sorafenib exhibited an epithelial-like morphology (Additional file 4: Figure S4A). These resistant cells significantly enhanced cell viability, and reduced ROS-associated cell apoptosis exposed to high doses of sorafenib (Additional file 4: Figure S4B–S4F). However, the FGF19 levels didn’t change in sorafenib-resistant cells compared with wildtype cells (Additional file 4: Figure S4**C**), indicating that sorafenib does not affect FGF19 expression levels.

**Fig. 5:**
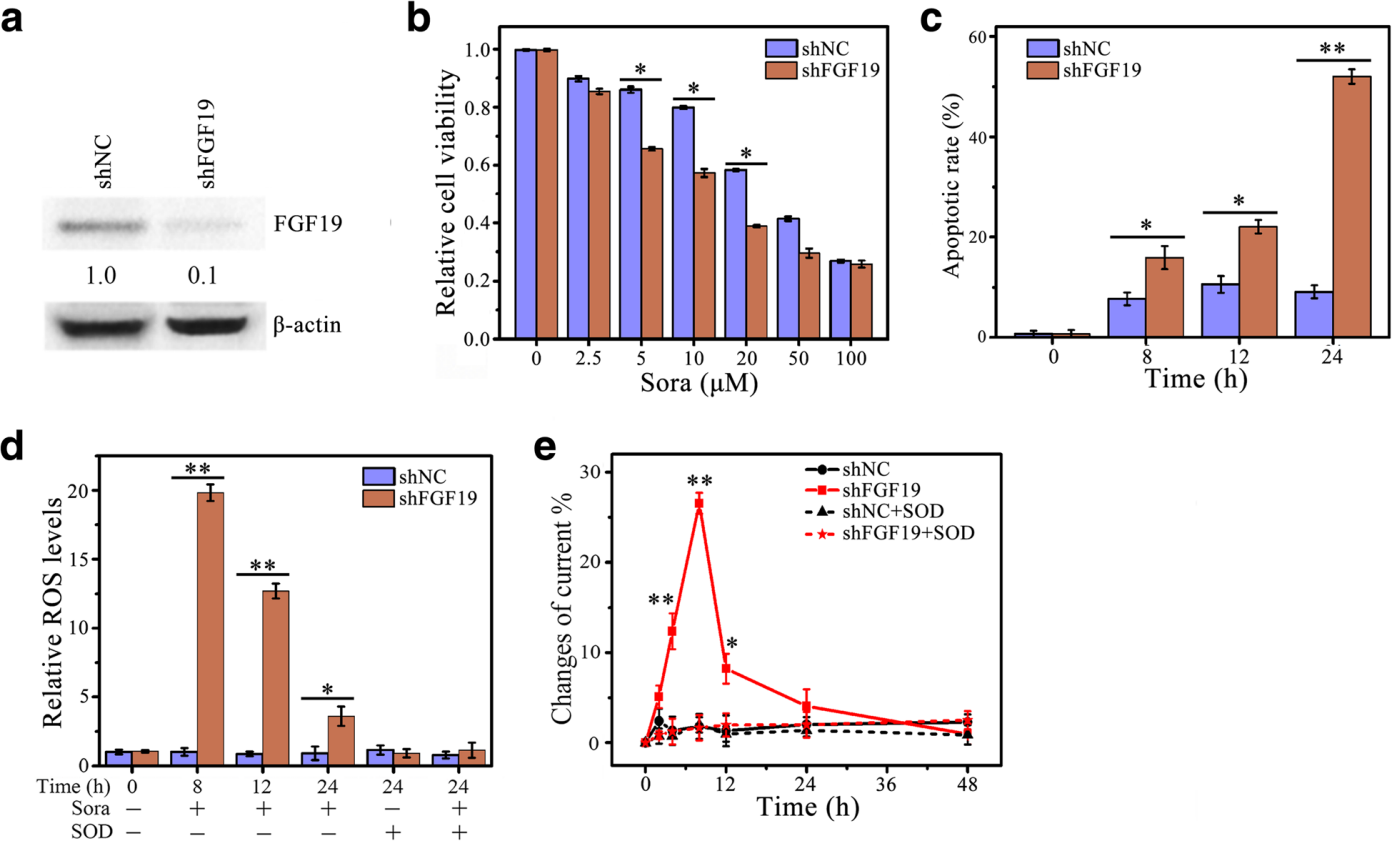
FGF19 Knockdown of in sorafenib-resistant HCC cells increases the sensitivity to sorafenib. (**a**) The knockdown effect of FGF19 in Sora-resistant MHCC97H (MHCC97H Sora-R) cells. (**b**-**e**) The effect of FGF19 knockdown on Sora-induced apoptosis in MHCC97H Sora-R cells. FGF19 was knocked down in MHCC97H Sora-R cells by lentiviral shRNA. FGF19 knockdown cells (shFGF19) and the control cells (shNC) were treated with different doses of Sora for 24 hours, and cell viability was determined by MTS assays (**b**). FGF19 knockdown cells (shFGF19) and the control cells (shNC) were treated with 20 μM of Sora over a series of time points before analysis. Apoptosis was determined by DAPI staining (**c**); ROS generation was determined by DCFH-DA staining (**d**); and O_2_^•−^ generation was determined by electrochemical biosensor (**e**). In **a**, expression levels were normalized against actin and reported relative to controls (fold changes shown below each lane). * *p* < 0.05; ** *p* < 0.01

We next determined the consequences of FGF19 depletion in the sorafenib-resistant cells exposed to high dose of sorafenib (20 μM) (Fig. 5 and Additional file 5: Figure S5). In sorafenib-resistant MHCC97H cells, knockdown of FGF19 (Fig. 5a) significantly enhanced the sensitivity to sorafenib, showing a decrease in cell viability (Fig. 5b) and an increase in ROS-associated cell apoptosis (Fig. 5c-e). The same phenotypes were observed in sorafenib-resistant HepG2 cells where FGF19 was depleted (Additional file 5: Figure S5). Taken together, our findings underlie the importance of FGF19 in sorafenib resistance and suggest that inactivation of FGF19 may have a potential therapeutic value in sorafenib treatment.

### Ponatinib facilitates sorafenib-resistant HCC cells to death

**Fig. 6:**
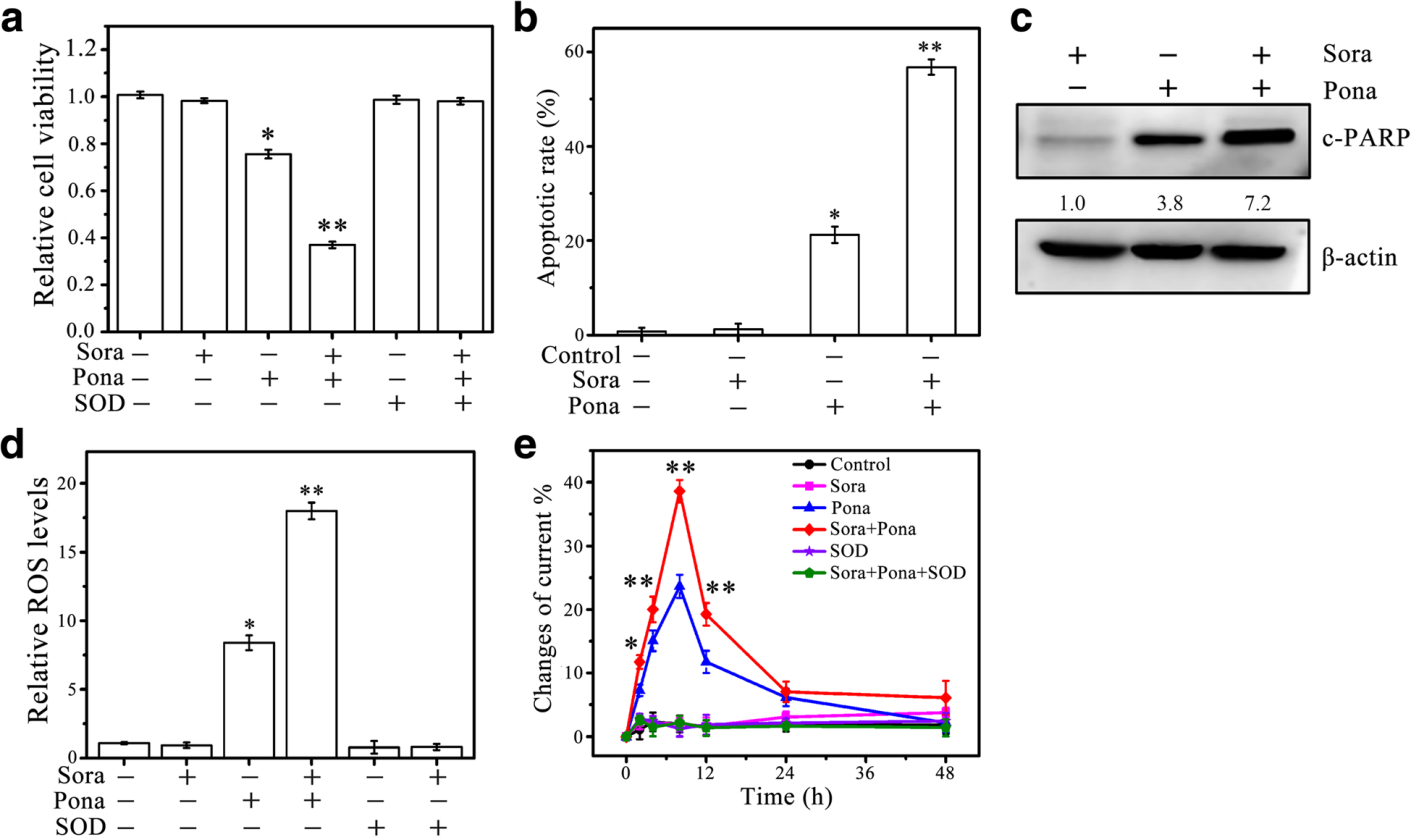
Ponatinib facilitates sorafenib-resistant HCC cells to death. **a-e** The effect of ponatinib (Pona) on Sora-induced ROS generation and apoptosis in Sora-resistant HCC cells. Sora-resistant MHCC97H (MHCC97H Sora-R) cells were treated with 20 µM of Sora and 10 µM of Pona singly or in combination. Cell viability was determined by MTS assays (**a**); apoptosis was determined by DAPI staining (**b**) and Western blot (**c**); ROS generation was determined by DCFH-DA staining (**d**); and O2•− generation was determined by electrochemical biosensor (**e**). In **c**, expression levels were normalized against actin and reported relative to controls (fold changes shown below each lane). * *p*<0.05; ** *p*<0.01

Our previous study have shown that ponatinib can effectively block FGF19/FGFR4 axis in HCC cells through suppression of FGFR4 activity [20]. We thus investigated the efficacy of ponatinib in sorafenib resistance. Treatment of sorafenib-resistant MHCC97H cells with 10 μM of ponatinib led to a 20% reduction in cell viability (Fig. 6a). Intriguingly, co-treatment of ponatinib (10 μM) and high dose of sorafenib (20 μM) showed a 60% decreased cell viability (Fig. 6a), suggesting that the synergistic effect of these two drugs is much greater than each drug given alone. Moreover, ponatinib facilitated sorafenib-resistant HCC cells to death which was associated with increased ROS, and co-treated with sorafenib enhanced these phenotypes (Fig. 6b–e). These data suggest that adjunct ponatinib could have additive anti-cancer effects when used with sorafenib in patients with HCC.

## Discussion

HCC is increasing in incidence with high fatality rate, and new therapies are urgently required to treat this disease. As first-line systemic therapy exists for patients with advanced HCC, sorafenib prolonged median survival and the time to progression of patients nearly 3 months [29, 30]. By dually targeting MAPK signaling and the activation of RTKs, sorafenib inhibits cell proliferation and induces cell apoptosis in HCC. However, the addiction switches and compensatory pathways are activated simultaneously or sequentially in the treatment of sorafenib, which may due to high molecular heterogeneity in HCC [31, 32]. Therefore, seeking novel anti-cancer agents or evaluating sorafenib in combination with other molecular targeted treatment is largely needed. A recent clinical report demonstrate that a copy number gain of FGF19 in HCC may represent a predictive biomarker for primary resistance to sorafenib [24]. In the present work, we provide new insights into the molecular basis of sorafenib resistance with the FGF19 involvement, and indicate that therapeutic strategies such as combining sorafenib with ponatinib can act synergistically to overcome the acquired resistance to sorafenib and improve anti-cancer effects in HCC.

ROS-sensitive signaling pathways are persistently elevated in many cancer types, where they participate in cell growth/survival, oxidative damage and metabolism [33, 34]. The half-period of free radical is only few seconds, therefore, detection of the short lifetime of free radicals particularly demands fast response of the analytical tool to the changes in concentration to obtain sufficient signal-to-noise ratios [35]. Electrochemical biosensors have become promising candidates for in situ analysis of free radicals [36]. We have successfully performed electrochemical biosensors to determine the intracellular oxidative balance in the PLX4032-treated melanoma cells [25]. Here, we explore that electrochemical biosensors combined with conventional methods of ROS detection can be used for monitoring extracellular and intracellular levels of oxidation/reduction more precisely during drug treatment.

Disproportional increase in intracellular ROS can induce cancer cell apoptosis [37], which can be achieved with sorafenib shown in this study, suggesting that sorafenib-induced high levels of ROS may turn on proapoptotic signaling. Recent studies indicate that FGF19/FGFR4 axis is a key signaling in certain forms of HCC [38, 39]. Interestingly, either knockdown of FGF19 or FGFR4 or treated with ponatinib enhances ROS levels and apoptosis in sorafenib-resistant HCC cells. These observations indicate that FGF19/FGFR4 axis also contributes to HCC resistance to sorafenib.

In our previous work, we have provided evidence that FGF19 secreted from either HCC cells or tumor microenvironment can activate its specific receptor FGFR4 on the surface of HCC cells [20]. We show here that the effects of sorafenib resistance can be overcome, at least partially, through blocking FGF19/FGFR4 signaling. Using the third-generation tyrosine-kinase inhibitor ponatinib, we found it was able to suppress almost all FGF19 activities through the inhibitory efficacy in FGFR4. We also show that in combination with the treatment of sorafenib, ponatinib plays a role in reversing the phenotypes induced by sorafenib resistance, such as reduced cell viability and enhanced cell apoptosis. These findings suggest the potential therapeutic effect of FGF19/FGFR4 blockade in patients with HCC, and demonstrate that the combination of ponatinib and sorafenib is more potent than either drug alone.

Knockdown of FGF19 leads to decreased cell proliferation, migration and invasion in HCC, which may also cause alternative signaling pathways that are either up- or down- regulated. Therefore, investigation of the overall gene expression profiles after FGF19 depletion will give us insight into the precise mechanism of FGF19-associated sorafenib resistance. Although our data are limited to in vitro characterization of HCC cells and will require further validation in animal models and clinical studies, this work provides a rational basis for FGF19/FGFR4 axis for the treatment of sorafenib-resistant HCC, and suggests that inhibition of FGF19/FGFR4 signaling may represent an attractive strategy for overcoming sorafenib resistance in HCC.

## Conclusions

In summary, this work demonstrates that elevated FGF19 expression or hyperactivation of FGF19/FGFR4 signaling in HCC cells is one of the main mechanisms of sorafenib resistance, and blocking FGF19/FGFR4 axis by ponatinib can overcome the resistance of HCC cells to sorafenib through enhancing ROS-associated apoptosis. Our studies provide the basis for developing a novel molecularly targeted therapeutics to prevent single drug resistance. In future work, we will collect the tumor samples from patients with sorafenib-sensitive or resistant HCC to explore clinical importance of the FGF19/FGFR4 axis.

## Additional files

Additional file 1: Figure S1. Representative images of sorafenib-induced ROS-associated cell apoptosis. HCC cell lines were treated with Sora (4 μM for MHCC97L, MHCC97H and SMCC-7721, and 6 μM for HepG2) over a series of time points. Apoptosis was determined by DAPI staining (A), and ROS generation was determined by DCFH-DA staining (B). (TIF 4418 kb)
Additional file 2: Figure S2. The structure diagram of electrochemical biosensors with a three-electrode system. (TIF 355 kb)
Additional file 3: Figure S3. FGFR4 overexpression does not chance sorafenib-induced cell response. (A–E) The effect of FGFR4 overxpression on Sora-induced HCC cell apoptosis and ROS generation. FGFR4 overexpressing MHCC97H cells (FGFR4 O/E) were treated with 4 μM of Sora over a series of time points before analysis. Cell viability was determined by MTS assays (A); cell apoptosis was determined by DAPI staining (B) and Western blot (C); ROS generation was determined by DCFH-DA staining (D); and O_2_^•−^ generation was determined by electrochemical biosensor (E). In (C), expression levels were normalized against actin and reported relative to controls (fold changes shown below each lane). (TIF 1164 kb)
Additional file 4: Figure S4. Sorafenib-resistant MHCC97H cells highly resistant to sorafenib-induced apoptosis and ROS generation. (A–F) The effect of Sora-resistant cells on Sora-induced HCC cell apoptosis and ROS generation. Sora-naive (WT) and Sora-resistant MHCC97H (MHCC97H Sora-R) cells were exposed to 20 μM of Sora over a series of time points before analysis. Morphological changes of cells were observed under microscope (A); cell viability was determined by MTS assays (B); apoptosis was determined by DAPI staining (C) and Western blot of c-PARP (D); ROS generation was determined by DCFH-DA staining (E); and O_2_^•−^ generation was determined by electrochemical biosensor (F). In C, expression levels were normalized against actin and reported relative to controls (fold changes shown below each lane).* *p* < 0.05; ** *p* < 0.01. 
Additional file 5: Figure S5. FGF19 knockdown in sorafenib-resistant HepG2 cells enhances ROS-associated apoptosis by sorafenib. (A) The knockdown effect of FGF19 in Sora-resistant HepG2 (HepG2 Sora-R) cells. (B–E) The effect of FGF19 knockdown on Sora-induced apoptosis in HepG2 Sora-R cells. FGF19 was knocked down in HepG2 Sora-R cells by lentiviral shRNA. FGF19 knockdown cells (shFGF19) and the control cells (shNC) were treated with different doses of Sora for 24 h. Cell viability was determined by MTS assays (B); apoptosis was determined by DAPI staining (C); ROS generation was determined by DCFH-DA staining (D), and O_2_^•−^ generation was determined by electrochemical biosensor (E). In A, expression levels were normalized against actin and reported relative to controls (fold changes shown below each lane). * *p* < 0.05; ** *p* < 0.01.

## Abbreviations

CV: Cyclic voltammetry
DAPI: 4′-6-diamidino-2-phenylindole
DCFH-DA: 2′, 7′-dichlorodihydrofluorescein diacetate
DMSO: Dimethyl sulfoxide
FGF19: Fibroblast growth factor 19
FGFR4: Fibroblast growth factor receptor-4
GSK3β: Glycogen synthase kinase 3β
HCC: Hepatocellular carcinoma
LI50: Lethal concentration 50%
MTS: 3-(4, 5-dimethylthiazol-2-yl)-5 (3-carboxymethonyphenol)-2-(4-sulfophenyl)-2H-tetrazolium
O_2_^•−^: Superoxide
ROS: Reactive oxidative species
RTKs: Receptor tyrosine kinases
SOD: Superoxide dismutase
