# Supplementary figures and images for: FGF19/FGFR4 signaling contributes to the resistance of hepatocellular carcinoma to sorafenib
Source: J Exp Clin Cancer Res. 2017 Jan 9;36:8. doi: 10.1186/s13046-016-0478-9 (PMC5223586; doi:10.1186/s13046-016-0478-9)

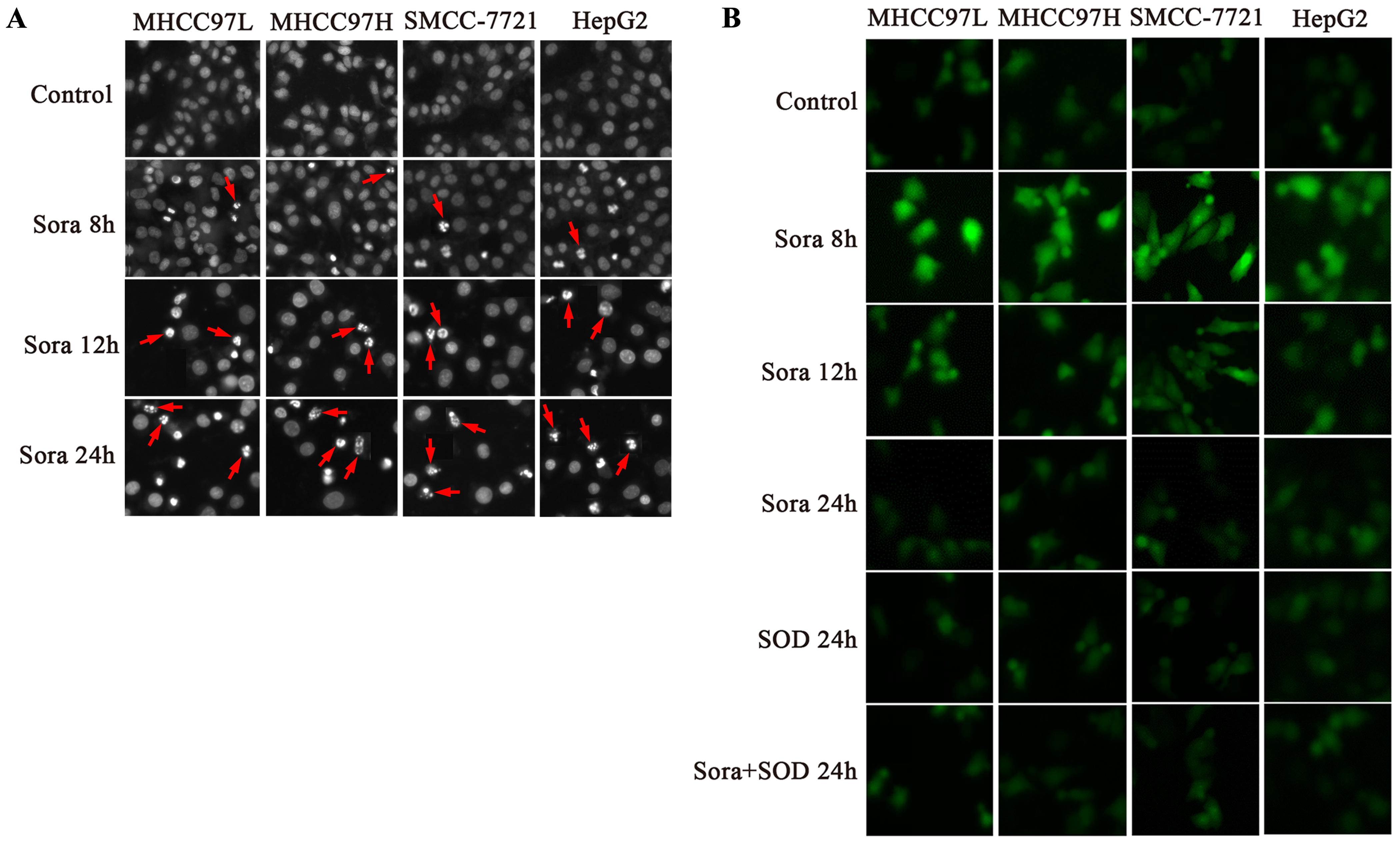

Supplement: Additional file 1: Figure S1. — Representative images of sorafenib-induced ROS-associated cell apoptosis. HCC cell lines were treated with Sora (4 μM for MHCC97L, MHCC97H and SMCC-7721, and 6 μM for HepG2) over a series of time points. Apoptosis was determined by DAPI staining (A), and ROS generation was determined by DCFH-DA staining (B). (TIF 4418 kb) [file 13046_2016_478_MOESM1_ESM.tif]

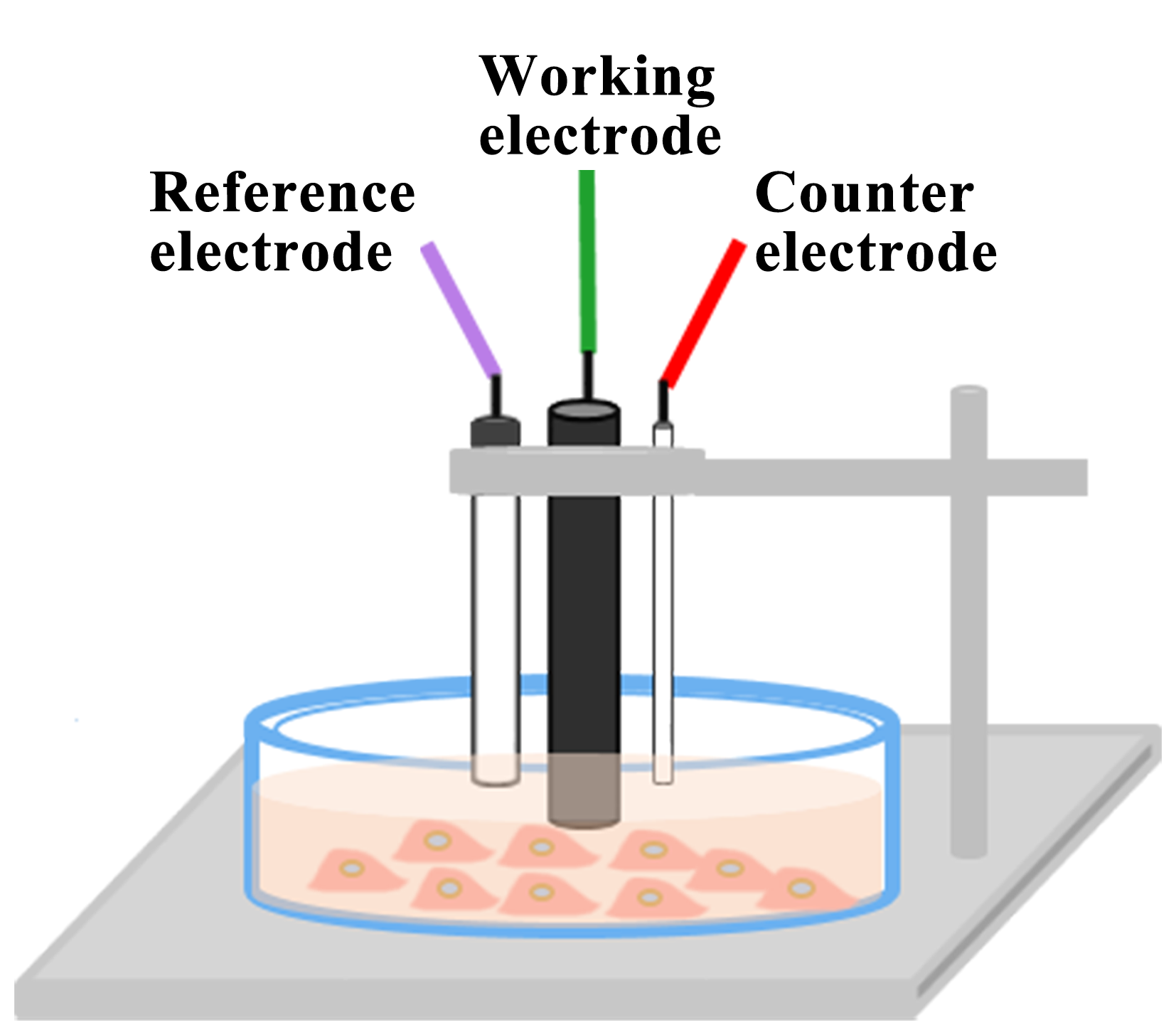

Supplement: Additional file 2: Figure S2. — The structure diagram of electrochemical biosensors with a three-electrode system. (TIF 355 kb) [file 13046_2016_478_MOESM2_ESM.tif]

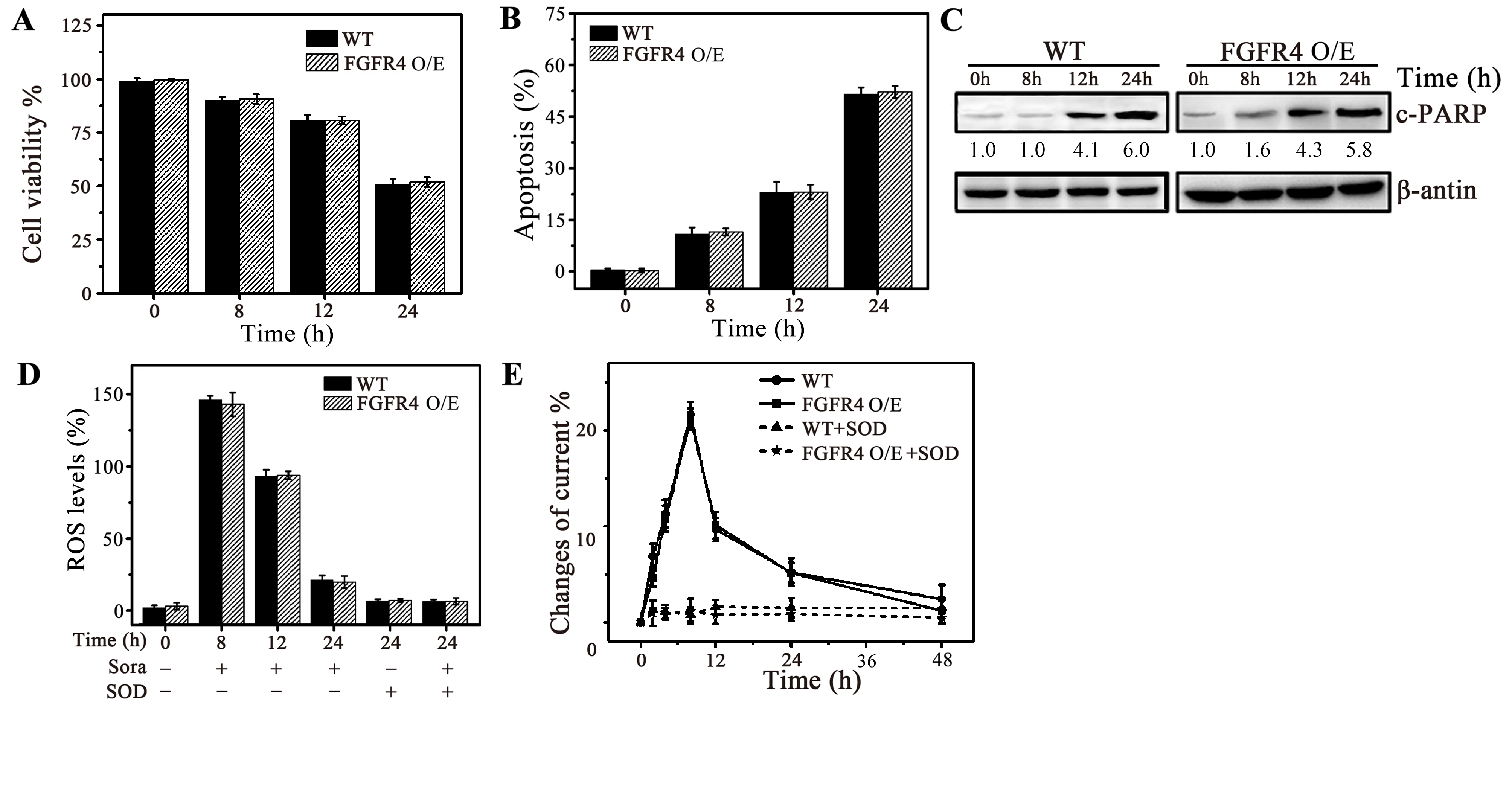

Supplement: Additional file 3: Figure S3. — FGFR4 overexpression does not chance sorafenib-induced cell response. (A–E) The effect of FGFR4 overxpression on Sora-induced HCC cell apoptosis and ROS generation. FGFR4 overexpressing MHCC97H cells (FGFR4 O/E) were treated with 4 μM of Sora over a series of time points before analysis. Cell viability was determined by MTS assays (A); cell apoptosis was determined by DAPI staining (B) and Western blot (C); ROS generation was determined by DCFH-DA staining (D); and O2 •− generation was determined by electrochemical biosensor (E). In (C), expression levels were normalized against actin and reported relative to controls (fold changes shown below each lane). (TIF 1164 kb) [file 13046_2016_478_MOESM3_ESM.tif]

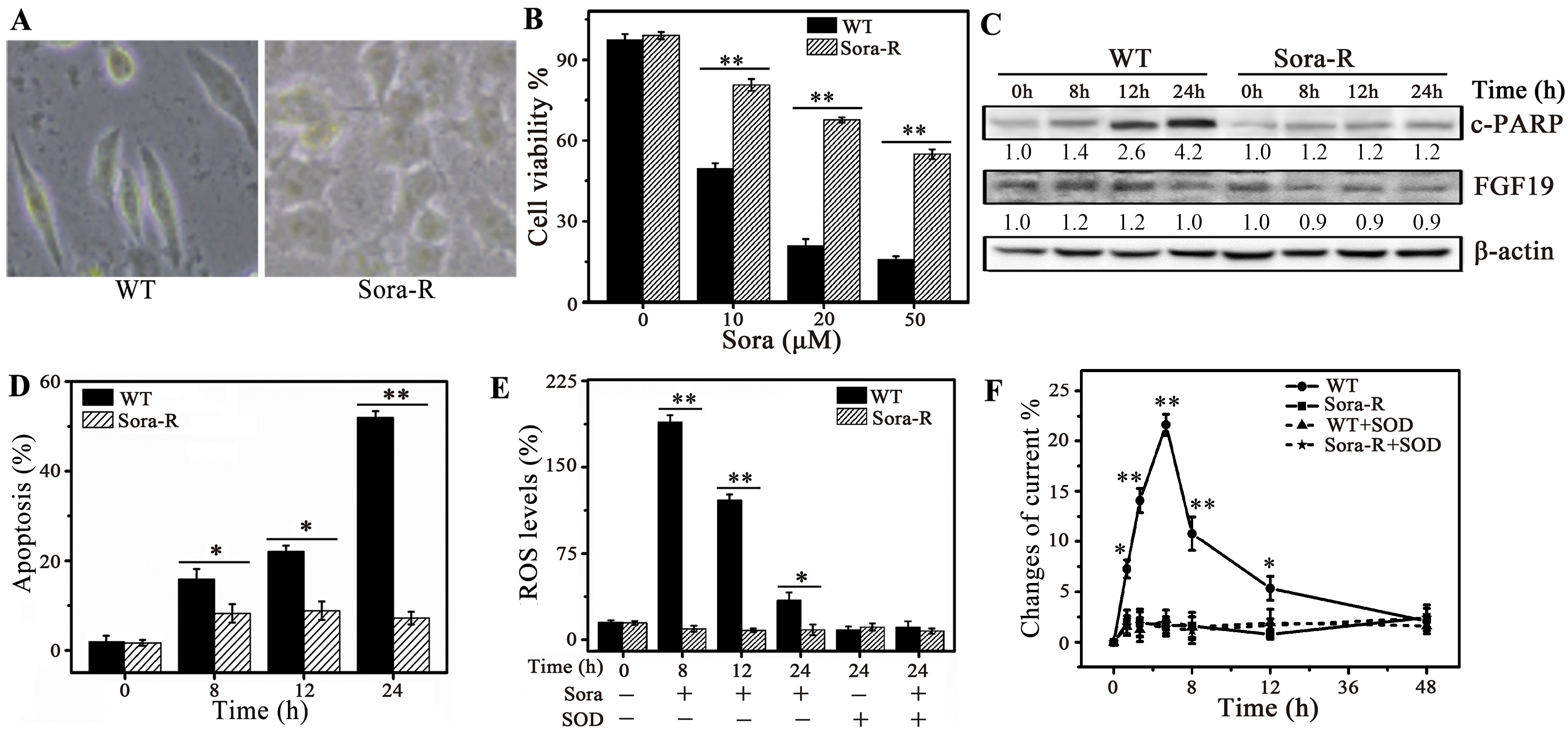

Supplement: Additional file 4: Figure S4. — Sorafenib-resistant MHCC97H cells highly resistant to sorafenib-induced apoptosis and ROS generation. (A–F) The effect of Sora-resistant cells on Sora-induced HCC cell apoptosis and ROS generation. Sora-naive (WT) and Sora-resistant MHCC97H (MHCC97H Sora-R) cells were exposed to 20 μM of Sora over a series of time points before analysis. Morphological changes of cells were observed under microscope (A); cell viability was determined by MTS assays (B); apoptosis was determined by DAPI staining (C) and Western blot of c-PARP (D); ROS generation was determined by DCFH-DA staining (E); and O2 •− generation was determined by electrochemical biosensor (F). In C, expression levels were normalized against actin and reported relative to controls (fold changes shown below each lane).* p < 0.05; ** p < 0.01. [file 13046_2016_478_MOESM4_ESM.tif]

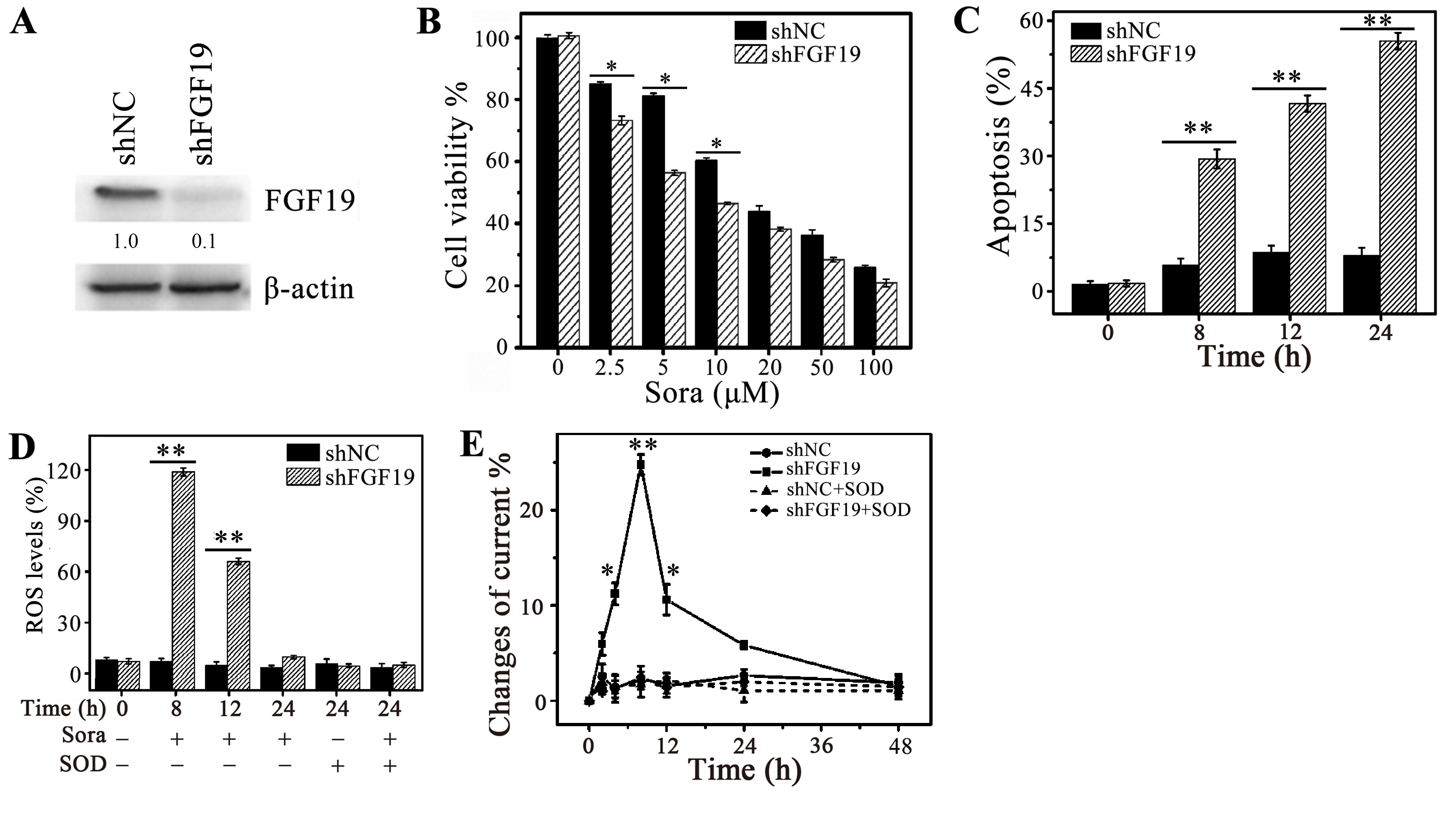

Supplement: Additional file 5: Figure S5. — FGF19 knockdown in sorafenib-resistant HepG2 cells enhances ROS-associated apoptosis by sorafenib. (A) The knockdown effect of FGF19 in Sora-resistant HepG2 (HepG2 Sora-R) cells. (B–E) The effect of FGF19 knockdown on Sora-induced apoptosis in HepG2 Sora-R cells. FGF19 was knocked down in HepG2 Sora-R cells by lentiviral shRNA. FGF19 knockdown cells (shFGF19) and the control cells (shNC) were treated with different doses of Sora for 24 h. Cell viability was determined by MTS assays (B); apoptosis was determined by DAPI staining (C); ROS generation was determined by DCFH-DA staining (D), and O2 •− generation was determined by electrochemical biosensor (E). In A, expression levels were normalized against actin and reported relative to controls (fold changes shown below each lane). * p < 0.05; ** p < 0.01. [file 13046_2016_478_MOESM5_ESM.tif]
